# Prevalence and determinants of perinatal depression among labour migrant and refugee women on the Thai-Myanmar border: a cohort study

**DOI:** 10.1186/s12888-020-02572-6

**Published:** 2020-04-15

**Authors:** Gracia Fellmeth, Emma Plugge, Mina Fazel, May May Oo, Mupawjay Pimanpanarak, Yuwapha Phichitpadungtham, Kerry Wai, Prakaykaew Charunwatthana, Julie A. Simpson, François Nosten, Raymond Fitzpatrick, Rose McGready

**Affiliations:** 1 Nuffield Department of Population Health, University of Oxford, L0/14, Richard Doll Building, Old Road Campus, Oxford, OX3 7LF UK; 2 Shoklo Malaria Research Unit, Mahidol-Oxford Tropical Medicine Research Unit, Faculty of Tropical Medicine, Mahidol University, 68/30 Bantung Road, Mae Sot, Tak 63110 Thailand; 3 Health and Justice Team, Health Improvement Directorate, Public Health England, 60 Caversham Road, London, Reading RG1 7EB UK; 4 Department of Psychiatry, University of Oxford, Warneford Hospital, Oxford, OX3 7JX UK; 5 Faculty of Tropical Medicine, Mahidol University, 420/6 Ratchawithi Road, Bangkok, 10400 Thailand; 6 Centre for Epidemiology and Biostatistics, Melbourne School of Population and Global Health, University of Melbourne, Melbourne, Australia; 7 Centre for Tropical Medicine and Global Health, Nuffield Department of Medicine, University of Oxford, South Parks Road, Oxford, OX1 3SY UK

**Keywords:** Perinatal, Depression, Migrant, Refugee, Low- and middle-income

## Abstract

**Background:**

Perinatal depression is a significant contributor to maternal morbidity and mortality globally. Migrant women, particularly those living in low- and middle-income settings, represent a particularly vulnerable group due to stressors experienced before, during and after migration. The vast majority of global migration flows occurring within and between low- and middle-income regions, yet existing evidence focuses predominantly on migrants in high-income destinations. This study aimed to redress this significant gap in the evidence by determining the prevalence and determinants of perinatal depression among migrant women on the Thai-Myanmar border.

**Methods:**

A cohort of labour migrant and refugee women was followed-up from the first trimester of pregnancy to one month post-partum. Depression status was assessed in the first, second and third trimesters of pregnancy and at one month post-partum using the Structured Clinical Interview for the Diagnosis of DSM-IV Disorders. Women diagnosed with depression had immediate access to care. Data on potential demographic, social and clinical associated factors was collected using a questionnaire. Prevalence and incidence of any depressive disorder and moderate-severe depressive disorder was calculated. Univariable and multivariable logistic regression using complete case analysis was used to estimate odds ratios (OR) of association between exposure variables and depression status.

**Results:**

Five hundred sixty-eight women participated. Period prevalence (from first trimester of pregnancy to one month post-partum) of moderate-severe perinatal depression was 18.5% (95% CI 15.4–21.9%). Overall, 15.4% (95% CI 11.8–19.6%) of women developed new-onset moderate-severe depression during the study period. Forty-two participants received treatment for depression. Risk factors were interpersonal violence (OR 4.5; 95% CI 1.9–11.1); history of trauma (OR 2.4; 95% CI 1.4–4.3); self-reported history of depression (OR 2.3; 95% CI 1.2–4.2); labour migrant status (OR 2.1; 95% CI 1.1–4.0); low social support (OR 2.1; 95% CI 1.1–3.7); and maternal age (OR 1.1 per year; 95% CI 1.0–1.1). Limitations of the study include that culturally specific manifestations of depression may have been missed.

**Conclusions:**

Perinatal depression represents a significant burden among migrant women on the Thai-Myanmar border. Programmes to address the determinants along with early case identification and effective treatment and referral systems are key to addressing perinatal depression in this low-resource setting.

## Background

Perinatal depression is a major contributor to maternal morbidity and mortality worldwide [1, 2]. Evidence consistently suggests that prevalence is higher in low- and middle-income countries (LMIC), with estimates ranging from 10.8–25.3% in LMIC compared with 7.4–19.2% in high-income countries (HIC) [1, 3, 4]. Untreated perinatal depression has been associated with a number of significant adverse outcomes including negative health behaviours in pregnancy [1], subsequent chronic and recurrent depression [1, 2], impaired ability to work and provide care, relationship breakdown [2, 5] and suicide – an important contributor to maternal deaths globally [6, 7]. In LMIC, perinatal depression is also associated with adverse child outcomes including low birthweight, stunting, insecure attachments and poor social development [5].

Risk factors for perinatal depression are multifactorial. Socio-economic disadvantage, low levels of support, interpersonal violence and prior psychopathology have been associated with perinatal depression across diverse settings [1, 2, 4, 8]. Migrant women constitute a particular risk group, experiencing many of the risk factors for perinatal depression prior to, during and following displacement [8–10]. Motherhood brings challenges of caring for a new infant, and doing this without adequate social support whilst navigating an unfamiliar culture and health system poses many challenges [10]. Although forced displacement is often associated with particular challenges, even planned migration can cause significant distress through separation from families and friends, isolation, lack of host language proficiency, discrimination and violence [10–12].

Meta-analyses have reported high pooled prevalence rates of perinatal depression ranging from 19 to 31% among migrant women [13, 14]. However, these figures stem from research which has focused largely on migrants living in HIC destinations. Less is known about migrant women who have resettled in LMIC, despite the fact that those who move from one LMIC setting to another constitute the majority of all migrants worldwide [15]. Though they may be less socially and culturally isolated due to remaining in closer proximity or migrating alongside family members, over the longer-term, migrant women who resettle in LMIC are likely to face poorer living conditions, greater socio-economic inequality and poorer access to resources in health and social care post-migration, compared with those resettling to HIC [11, 15].

The significant impacts of perinatal depression coupled with ever-increasing global migration flows and severe treatment gaps for mental disorders in LMIC render the perinatal mental health of migrant women in low-income settings an urgent global health priority. There is a pressing need for reliable data on the prevalence of perinatal depression and context-specific risk factors in LMIC. Combined with appropriate identification and management mechanisms, an improved understanding of the condition can improve women’s experiences of pregnancy and the postpartum period.

## Methods

### Aim

This study aimed to determine the prevalence of, and factors associated with, perinatal depression among labour migrant and refugee women living on the Thai-Myanmar border.

### Setting

Decades of conflict in Myanmar have resulted in large-scale displacement of an estimated 200,000 labour migrants and 145,000 refugees to the Thai-Myanmar border [16]. Refugees live in established camps on the Thai side of the border where they receive a basic package of aid including healthcare, food rations and housing. Though the camps provide security, refugees’ opportunities for employment are limited and movement in and out of the camps is highly restricted. By contrast, labour migrants live in rural villages on both sides of the border and seek employment in agriculture, manufacturing and service industries where they receive wages significantly below Thailand’s legal minimum wage [12]. Many labour migrants lack formal documentation, rendering them vulnerable to exploitation and deportation. Labour migrants lack entitlement to health and education in Thailand, contrasting with refugees for whom such services are much more accessible.

Mental health remains a neglected area on the Thai-Myanmar border due to stigmatisation and a low awareness among health professionals and the general community. Furthermore, the small number of studies exploring the mental health of this population have yielded conflicting findings. One study of Karenni refugees in Northern Thailand found a 41% prevalence of depression symptoms [17], while in a more recent study only 5 % of displaced adults in Karen State, Myanmar, screened positive for depression [18]. These differences highlight the uncertainty around depression estimates. Suicide is a significant contributor to maternal mortality in this region, accounting for 9% of maternal deaths between 1998 and 2015 [7]. Despite this, perinatal depression has not been examined in this setting.

**Fig. 1:**
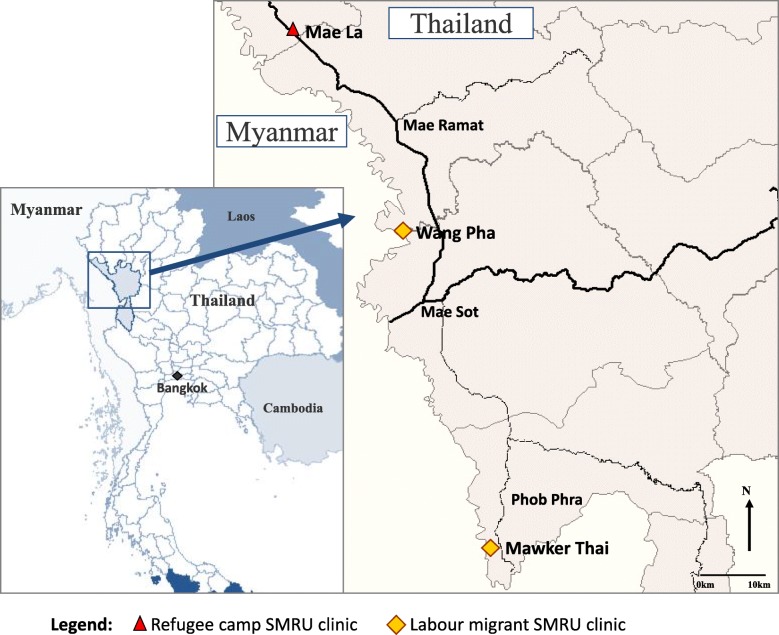
Map of refugee and labour migrant clinics provided by Shoklo Malaria Research Unit

The Shoklo Malaria Research Unit (SMRU) is a research collaboration between the Mahidol-Oxford Tropical Medicine Research Unit (Thailand), Oxford University (UK) and the Wellcome Trust (UK). SMRU has carried out research and provided maternity services on the Thai-Myanmar border since 1986 at three clinic sites serving the refugee population in Maela camp and labour migrants at Mawker Tai and Wang Pha (Fig. 1).

### Study design

Methods of this cohort study have been described in detail elsewhere [19]. Participants were labour migrant and refugee women attending SMRU antenatal clinics (ANC) at Mawker Tai (labour migrants), Wang Pha (labour migrants) and Maela (refugees) between October 2015 and April 2016. Women were eligible if they were aged ≥18 years and in their first trimester of pregnancy (estimated gestational age (EGA) as determined by ultrasound scan < 14 weeks). Verbal and written explanations were provided in the three main local languages: Burmese, Sgaw Karen and Poe Karen. Women who agreed to participate provided written informed consent. Women were followed up from their first trimester of pregnancy until one month post-partum, with data collection occurring at four time points: in the first, second and third trimesters of pregnancy and at one month post-partum. In this setting, women attend antenatal clinics held at SMRU health clinics frequently throughout their pregnancy. As a result, women have a high level of contact with healthcare professionals and receive good quality care during this time.

Data was collected by a physician and trained staff members of SMRU. The SMRU staff are themselves refugees and migrants who have undergone training at SMRU to become healthcare workers. They therefore have much insight into the situations of women attending the clinic and are sensitive to the cultural factors at play. The SMRU staff conducting this study were fluent in Burmese, Karen and English and had extensive experience of working with the local population.

**Fig. 2:**
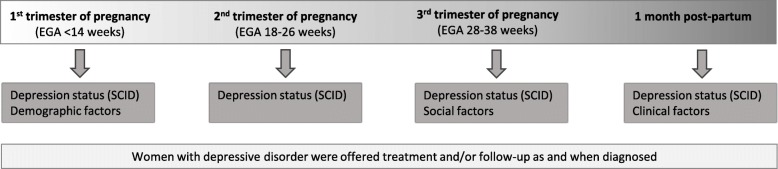
Timeline of data collection during 1st, 2nd and 3rd trimesters of pregnancy and one month post-partum. EGA estimated gestational age; SCID Structured Clinical Interview for the Diagnosis of DSM-IV Disorders

Depression status was assessed at four timepoints: in the first (any time before EGA 14 weeks), second (any time between EGA 18–26 weeks) and third (any time between EGA 28–38 weeks) trimesters of pregnancy and at one month post-partum using the *Structured Clinical Interview for the Diagnosis of DSM-IV Disorders* (SCID). The timing of data collection is summarised in Fig. 2. Diagnostic categories assessed for were Major Depressive Disorder, Minor Depressive Disorder and Depressive Disorder ‘Not Otherwise Specified’ (NOS). All interviewers had received training in administering the SCID prior to the start of the study. SCID responses were independently scored by the study physician and an independent physician. Any discrepancies which could not be resolved by the two physicians were discussed with a psychiatrist and a senior research officer in psychiatry until a consensus could be reached. Women who were diagnosed as having depressive disorder were reviewed by a physician and offered treatment in the form of counselling and/or medication as appropriate as soon as they were diagnosed as having the condition. Those with severe symptoms and those who were considered to be at risk of harming themselves or others were admitted for observation. Questionnaires designed by the researchers were used to collect data on demographic factors (first trimester) and social factors (third trimester). Data on clinical factors was collected using participants’ electronic medical records (held by SMRU) at 1 month post-partum. The presence of interpersonal violence was assessed using the single, direct question: “Does your partner ever hurt, hit or threaten you?” and was scored as a binary item, with 0 and 1 representing the absence and presence of any interpersonal violence, respectively. In order to assess the concept of social support, we chose a question on the perceived sufficiency of support, which has been identified as an important factor in perinatal well-being that is often independent of actual received support [20]. A single, direct question was used: “Do you feel the support you receive is enough?”. This question was scored as a binary item, with 0 indicating insufficient support and 1 indicating sufficient support.

### Statistical analysis

For statistical analysis, the outcome was defined in two ways. First, analyses were run using the outcome ‘*moderate-severe depression’*, defined as the presence of either Major Depressive Disorder or Minor Depressive Disorder at any one timepoint during the study period (i.e. at least once in either the first, second or third trimester of pregnancy or at one month post-partum). Second, analyses were run using the outcome ‘*any depression*’, defined as the presence of either Major Depressive Disorder, Minor Depressive Disorder or Depressive Disorder NOS at any one timepoint during the study period. The target sample size was 500, based on an estimated prevalence of perinatal depression of 15%, an assumed 20% loss to follow-up over one year, 80% power and 95% confidence interval to detect associations of 2.5-fold in magnitude [21]. Point prevalence was calculated for each timepoint of data collection, i.e. for each trimester of pregnancy and at one month post-partum. Period prevalence was calculated for study period, i.e. from the first trimester of pregnancy through to one month post-partum. Incidence for the study period was defined as the number of new cases arising over the duration of the study amongst those who did not have depression at study initiation in the first trimester.

Logistic regression using complete case analysis was used to estimate odds ratios (OR) of association between exposure variables and depression status. In the univariable analysis, *p*-values of < 0.10 were considered to demonstrate statistically significant associations between exposure variables and the outcome. These variables as well as any variables considered to be clinically important were entered into a multivariable logistic regression model using a forward stepwise approach following Hosmer and Lemeshow’s method of purposeful selection of covariates [22]. Covariates that improved the fit of the model on likelihood ratio testing (LR Chi^2^) were retained while those that did not were discarded. The multivariable model included only those participants for whom none of the variables included in the model were missing. In the final multivariable model, factors with a *p*-value of < 0.05 were considered to be statistically significantly associated with depression. Analyses were conducted in *STATA 13*.

## Results

A total of 567 (317 labour migrant and 250 refugee) women participated. The baseline participant characteristics are reported elsewhere [19]. The median age was 25 years. Refugees were predominantly of Sgaw Karen ethnicity, while among labour migrants the predominant ethnicity was Burman. Women had completed a median of four years of formal education. The main employment sector was agricultural work (39.2%), although over a third of participants were not in paid employment (35.7%). Table 1 shows the point prevalence, period prevalence and incidence of perinatal depression. The period prevalence of moderate-severe depression and any perinatal depression were 18.5% (95% CI 15.4–21.9%) and 39.8% (95% CI 35.7–43.9%), respectively. Among women who completed all four assessments, 350 did not have moderate-severe depression at baseline; of these, 15.4% (95% CI 11.8–19.6%) developed new onset moderate-severe depression during the study period. Among those who did not have any depression at baseline, the incidence of any depression during the course of the study period was 28.5% (95% CI 23.3–34.2).

**Table 1:** Prevalence and incidence of any and moderate-severe perinatal depression

	Any depression		Moderate-severe depression	
	No. of cases	Proportion (95% CI)	No. of cases	Proportion (95% CI)
Prevalence				
1st trimester	146/567	25.7 (22.2–29.6)	46/567	8.1 (6.0–10.7)
2nd trimester	103/481	21.4 (17.8–25.4)	37/481	7.7 (5.5–10.4)
3rd trimester	91/460	19.8 (16.2–23.7)	23/460	5.0 (3.2–7.4)
1 m post-partum	78/396	19.7 (15.9–24.0)	33/396	8.3 (5.8–11.5)
Period prevalence^a^	226/567	39.8 (35.7–43.9)	105/567	18.5 (15.4–21.9)
Incidence				
2nd trimester	39/360^1^	10.8 (7.8–14.5)	24/449^4^	5.3 (3.5–7.8)
3rd trimester	19/293^2^	6.8 (4.2–10.3)	11/392^5^	2.8 (1.4–5.0)
1 m post-partum	18/224^3^	8.0 (4.8–12.4)	17/317^6^	5.4 (3.2–8.4)
Overall incidence^b^	79/277	28.5 (23.3–34.2)	54/350	15.4 (11.8–19.6)

^a^ Women with depression on at least one occasion, as a proportion of all participating women

^b^ Analysis restricted to women who completed all 4 assessments

^1^ Analysis restricted to women with no depression in 1st trimester

^2^ Analysis restricted to women with no depression in 1st or 2nd trimester

^3^ Analysis restricted to women with no depression in 1st, 2nd or 3rd trimester

^4^ Analysis restricted to women with no moderate-severe depression in 1st trimester

^5^ Analysis restricted to women with no moderate-severe depression in 1st or 2nd trimester

^6^ Analysis restricted to women with no moderate-severe depression in 1st, 2nd or 3rd trimester

Table 2 shows the persistence of perinatal depression. Of those women with moderate-severe depression in the first trimester, none remained depressed at one month post-partum. Of those with any depression in the first trimester, 11.0% (95% CI 6.4–17.2%) remained depressed at one month post-partum.

**Table 2:** Persistence of perinatal depression from the first trimester of pregnancy to 1 month post-partum

1st trimester	Women with persisting depression^a^					
	2nd trimester		3rd trimester		1 m post-partum	
	n	Proportion (95% CI)	n	Proportion (95% CI)	n	Proportion (95% CI)
Any depression (*n* = 146)	64	43.8 (35.6–52.3)	32	21.9 (15.5–29.5)	16	11.0 (6.4–17.2)
Moderate-severe depression (*n* = 46)	10	21.7 (10.9–36.4)	2	4.3 (0.5–14.8)	0	0.0 (0.0–7.7)^b^

^a^ Women with persisting depression as a proportion of those depressed in 1st trimester

^b^ One-sided (97.5%) confidence interval

During the study period, 42 participants were referred for treatment for depression, all of whom agreed. Of these, 22 women received counselling and sertraline, and 20 received counselling alone. In the counselling and sertraline group, 19 women returned for follow-up of whom 15 (79%) improved, 2 (10.5%) remained the same and 2 (10.5% deteriorated). In the counselling only group, 10 (50%) improved, 6 (30%) remained the same and 4 (20%) deteriorated.

Table 3 shows univariable and multivariable associations between socio-demographic and clinical exposure variables and moderate-severe depression. A total of 444 women in the ‘moderate-severe depression’ group had no missing data and were included in multivariable analysis. In the multivariable analysis, the following variables remained statistically significantly associated with moderate-severe depression after adjustment for all other variables in the model: interpersonal violence (OR 4.5; 95% CI 1.9–11.1); history of trauma (OR 2.4; 95% CI 1.4–4.3); self-reported history of depression (OR 2.3; 95% CI 1.2–4.2); labour migrant (as compared with refugee) status (OR 2.1; 95% CI 1.1–4.0); perceived insufficiency of social support (OR 2.1; 95% CI 1.1–3.7); and maternal age (OR 1.1; 95% CI 1.0–1.1).

**Table 3:** Univariable and multivariable associations between socio-demographic and clinical factors and moderate-severe perinatal depression (*n* = 444)

	Moderate-severe depression n/N (%)	No moderate-severe depression n/N (%)	Unadjusted OR (95% CI)	*p*-value	Adjusted OR* (95% CI)	*p*-value
Age (yrs)^†^	28 [18–45]	25 [18–50]	1.06 (1.03–1.09)	**< 0.01**	1.05 (1.01–1.09)	**0.02**
**Ethnicity**						
Burman	35/161 (21.7)	126/161 (78.3)	1.00	0.33	–	–
Sgaw Karen	43/278 (15.5)	235/278 (84.5)	0.66 (0.40–1.08)			
Poe Karen	13/66 (19.7)	53/66 (80.3)	0.88 (0.43–1.80)			
Other	14/63 (22.2)	49/63 (77.8)	1.03 (0.51–2.08)			
**Language**						
Burmese	51/239 (21.3)	188/239 (78.7)	1.00	0.16		
Sgaw Karen	43/280 (15.4)	237/280 (84.6)	0.67 (0.43–1.05)		–	–
Other	11/49 (22.4)	38/49 (77.6)	1.07 (0.51–2.23)			
**Religion**						
Buddhist	77/408 (18.9)	331/408 (81.1)	1.00	0.81	–	–
Christian	19/115 (16.5)	96/115 (83.5)	0.85 (0.49–1.48)			
Muslim	9/45 (20.0)	36/45 (80.0)	1.07 (0.50–2.32)			
**Education**						
Under 3 years	53/255 (20.8)	202/255 (79.2)	1.00	0.15	–	–
3 to 6 years	32/164 (19.5)	132/164 (80.5)	0.92 (0.57–1.51)			
7 or more years	19/143 (13.3)	124/143 (86.7)	0.58 (0.33–1.03)			
**Literacy**						
Yes	69/392 (17.6)	323/392 (82.4)	1.00	0.42	–	–
No	36/176 (20.5)	140/176 (79.5)	1.20 (0.77–1.89)			
**Employment**						
Employed	69/348 (19.8)	279/348 (80.2)	1.00	0.74	–	–
Unemployed	36/193 (18.7)	157/193 (81.3)	0.93 (0.59–1.45)			
**Other household member works**						
Yes	70/369 (19.0)	299/369 (81.0)	1.00	0.55	–	–
No	20/92 (21.7)	72/92 (78.3)	1.19 (0.18–0.30)			
**Telephone ownership**						
Yes	51/329 (15.5)	278/329 (84.5)	1.00	**0.03**	–	–
No	54/239 (22.6)	185/239 (77.4)	1.59 (1.04–2.43)			
**Site**						
Maela (refugee)	49/250 (19.6)	201/250 (80.1)	1.00	**< 0.01**	–	–
Wang Pha (migrant)	39/155 (25.2)	116/155 (74.8)	1.38 (0.85–2.23)			
Mawker Tai (migrant)	17/163 (10.4)	146/163 (89.6)	0.48 (0.26–0.86)			
**Migrant status**						
Refugee	49/250 (19.6)	201/250 (80.4)	1.00	0.55	1.00	**0.03**
Labour migrant	56/318 (17.6)	262/318 (82.4)	0.88 (0.57–1.34)		2.08 (1.09–4.00)	
**Duration since migration**						
≤ 1 year	18/64 (28.1)	46/64 (71.9)	1.00	**0.01**	–	–
> 1 year	18/138 (13.0)	120/138 (87.0)	0.38 (0.18–0.80)			
**Alcohol**						
No	99/543 (18.2)	444/543 (81.8)	1.00	0.48	–	–
Yes	6/25 (24.0)	19/25 (76.0)	1.42 (0.55–3.64)			
**Smoking**						
No	90/512 (17.6)	422.512 (82.4)	1.00	0.11	–	–
Yes	15/56 (26.8)	41/56 (73.2)	1.72 (0.91–3.23)			
**Chew betel nut**						
No	46/317 (14.5)	271/317 (85.5)	1.00	**< 0.01**	–	–
Yes	59/251 (23.5)	192/251 (76.5)	1.81 (1.18–2.78)			
**Self-reported depression history**						
No	58/419 (13.8)	361/419 (86.2)	1.00	**< 0.01**	1.00	**0.01**
Yes	46/147 (31.3)	101/147 (68.7)	2.83 (1.82–4.43)		2.25 (1.21–4.17)	
**Sufficient social support**						
Yes	51/320 (15.9)	269/320 (84.1)	1.00	**< 0.01**	1.00 2.07 (1.14–3.73)	**0.02**
No	38/137 (27.7)	99/137 (72.3)	2.02 (1.25–3.27)			
**Trauma**						
None	26/239 (10.9)	213/239 (89.1)	1.00	**< 0.01**	1.00	**< 0.01**
≥ 1 trauma event	79/329 (24.0)	250/329 (76.0)	2.59 (1.60–4.18)		2.43 (1.38–4.30)	
**Interpersonal violence**						
No	74/430 (17.2)	356/430 (82.8)	1.00	**< 0.01**	1.00	**< 0.01**
Yes	15/27 (55.6)	12/27 (44.4)	6.01 (2.70–13.37)		4.54 (1.86–11.11)	
**Maternal BMI**^a^						
Normal	56/296 (18.9)	240/296 (81.1)	1.00	0.96	–	–
Underweight	18/99 (18.2)	81/99 (81.8)	0.95 (0.53–1.71)			
Overweight/obese	31/173 (17.9)	142/173 (82.1)	0.94 (0.58–1.52)			
**Primigravid**						
Primigravida	19/160 (11.9)	141/160 (88.1)	1.00	**< 0.01**	–	–
Multigravida	86/408 (21.1)	322/408 (78.9)	1.98 (1.16–3.38)			
**Pregnancy**						
Planned	49/310 (15.8)	261/310 (84.2)	1.00	**< 0.01**	–	–
Unplanned	38/144 (26.4)	106/144 (73.6)	1.91 (1.18–3.09)			
**Mode of delivery**						
Normal	81/411 (19.7)	330/411 (80.3)	1.00	0.91	–	–
Instrumental	9/44 (20.5)	35/44 (79.5)	1.05 (0.48–2.27)			

Bold denotes statistical significance: *p* < 0.10 in univariable and *p* < 0.05 in multivariable analysis

^†^ Results presented as median [range]

*Adjusted odds ratios presented only for variables which remained significantly associated with moderate-severe perinatal depression in multivariable logistic regression. Only participants for whom no data was missing were included in multivariable analysis

^a^ Assessed in first trimester of pregnancy

Table 4 shows results for analyses conducted using any depression as the outcome. A total of 400 women in the ‘any depression’ group had no missing data and were included in the multivariable analysis. In this analysis, five variables remained significantly associated with the outcome in the multivariable model: interpersonal violence (OR 4.4; 95% CI 1.5–12.5); self-reported history of depression (OR 3.4; 95% CI 2.0–5.6); history of trauma (OR 2.2; 95% CI 1.4–3.4); not owning a telephone (OR 1.6; 95% CI 1.0–2.5); and the presence of another working household member (OR 0.6; 95% CI 0.3–1.0).

**Table 4:** Univariable and multivariable associations between socio-demographic and clinical factors and any perinatal depression (*n* = 400)

	Any depression n/N (%)	No depression n/N (%)	Unadj. OR (95% CI)	*p*-value	Adjusted OR* (95% CI)	*p*-value
**Age** (yrs)^†^	26 [18–50]	25 [18–45]	1.04 (1.01–1.07)	**< 0.01**	**–**	**–**
**Ethnicity**						
Burman	63/161 (39.1)	98/161 (60.9)	1.00	**0.06**	–	–
Sgaw Karen	100/278 (36.0)	178/278 (64.0)	0.87 (0.59–1.30)			
Poe Karen	29/66 (43.9)	37/66 (56.1)	1.22 (0.68–2.18)			
Other	34/63 (54.0)	29/63 (46.0)	1.82 (1.01–3.28)			
**Language**						
Burmese	103/239 (43.1)	136/239 (56.9)	1.00	**0.04**	–	–
Sgaw Karen	98/280 (35.0)	182/280 (65.0)	0.71 (0.50–1.01)			
Other	25/49 (51.0)	24/49 (49.0)	1.38 (0.74–2.55)			
**Religion**						
Buddhist	154/408 (37.8)	254/408 (62.2)	1.00	**0.02**	–	–
Christian	45/115 (39.1)	70/115 (60.9)	1.06 (0.69–1.62)			
Muslim	27/45 (60.0)	18/45 (40.0)	2.47 (0.32–4.64)			
**Education**						
Under 3 years	107/255 (42.0)	148/255 (58.0)	1.00	0.47	–	–
3 to 6 years	65/164 (39.6)	99/164 (60.4)	0.91 (0.61–1.35)			
7 or more years	51/143 (35.7)	92/143 (64.3)	0.77 (0.50–1.17)			
**Literacy**						
Yes	155/392 (39.5)	237/392 (60.5)	1.00	0.86	–	–
No	71/176 (40.3)	105/176 (59.7)	1.03 (0.72–1.49)			
**Employment status**						
Employed	139/348 (39.9)	209/348 (60.1)	1.00	0.42	–	–
Unemployed	84/193 (43.5)	109/193 (56.5)	1.16 (0.81–1.65)			
**Other household member works**						
Yes	148/369 (40.1)	221/369 (59.9)	1.00	**0.02**	1.00	**0.04**
No	49/92 (53.3)	43/92 (46.7)	1.70 (0.07–2.69)		0.57 (0.33–0.98)	
**Telephone**						
Yes	121/329 (36.8)	208/329 (63.2)	1.00	**0.09**	1.00	**0.04**
No	105/239 (43.9)	134/239 (56.1)	1.35 (0.96–1.89)		1.59 (1.01–2.48)	
**Site**						
Maela (refugee)	114/250 (45.6)	136/250 (54.4)	1.00	**< 0.01**	–	–
Wang Pha (migrant)	66/155 (42.6)	89/155 (57.4)	0.88 (0.59–1.33)			
Mawker Tai (migrant)	46/163 (28.2)	117/163 (71.8)	0.47 (0.31–0.72)			
**Migrant status**						
Refugee	114/250 (45.6)	136/250 (54.4)	1.00	**0.01**	–	–
Labour migrant	112/318 (35.2)	206/318 (64/8)	0.65 (0.46–0.91)			
**Duration since migration**						
≤ 1 year	32/64 (50.0)	32/64 (50.0)	1.00	**< 0.01**	–	–
> 1 year	35/138 (25.4)	103/138 (74.6)	0.34 (0.18–0.63)			
**Alcohol**						
No	215/543 (39.6)	328/543 (60.4)	1.00	0.66	–	–
Yes	11/25 (44.0)	14/25 (56.0)	1.20 (0.53–2.69)			
**Smoking**						
No	202/512 (39.5)	310/512 (60.5)	1.00	0.62	–	
Yes	24/56 (42.9)	32.56 (57.1)	1.15 (0.66–2.01)			
**Chew betel nut**						
No	116/317 (36.6)	201/317 (63.4)	1.00	**0.08**	–	–
Yes	110/251 (43.8)	141/251 (56.2)	1.35 (0.96–1.90)			
**Self-reported depression history**						
No	130/419 (31.0)	289/419 (69.0)	1.00	**< 0.01**	1.00	**< 0.01**
Yes	95/147 (64.6)	52/147 (35.4)	4.06 (2.73–6.04)		3.36 (2.01–5.61)	
**Sufficient social support**						
Yes	120/320 (37.5)	200/320 (37.5)	1.00	**< 0.01**	–	–
No	76/137 (55.5)	61/137 (44.5)	2.08 (1.37–3.12)			
**Trauma**						
None	69/239 (28.9)	170/239 (71.1)	1.00	**< 0.01**	1.00	**< 0.01**
≥ 1 trauma	157/329 (47.7)	172/329 (52.3)	2.25 (1.58–3.20)		2.17 (1.38–3.41)	
**Interpersonal violence**						
No	174/430 (40.5)	256/430 (59.5)	1.00	**< 0.01**	1.00	**< 0.01**
Yes	22/27 (81.5)	5/27 (18.5)	6.47 (2.41–17.42)		4.36 (1.52–12.50)	
**Maternal BMI**^**a**^						
Normal	121/296 (40.9)	175/296 (59.1)	1.00	0.86	–	–
Underweight	38/99 (38.4)	61/99 (61.6)	0.90 (0.56–1.44)			
Overweight/obese	67/173 (38.7)	106/173 (61.3)	0.91 (0.62–1.34)			
**Primigravid**						
Primigravida	48/160 (30.0)	112/160 (70.0)	1.00	**< 0.01**	–	–
Multigravida	178/408 (43.6)	230/408 (56.4)	1.81 (1.22–2.67)			
**Pregnancy**						
Planned	115/310 (37.1)	195/310 (62.9)	1.00	**< 0.01**	–	–
Unplanned	77/144 (53.5)	67/144 (46.5)	1.95 (1.31–2.91)			
**Mode of delivery**						
Normal	178/411 (43.4)	233/411 (56.7)	1.00	0.55	–	–
Instrumental	17/44 (38.6)	27/44 (61.4)	0.82 (0.43–1.56)			

Bold denotes *p* < 0.10 in univariable and *p* < 0.05 at multivariable analysis

^†^ Results presented as median [range]

*Adjusted odds ratios presented only for variables which remained significantly associated with any perinatal depression in multivariable logistic regression. Only participants for whom no data was missing were included in multivariable analysis

^a^ Assessed in first trimester of pregnancy

The association between antenatal and postnatal depression was examined independently. After controlling for all other factors, moderate-severe antenatal depression was significantly associated with moderate-severe postnatal depression (OR 5.0; 95% CI 2.1–12.3). Using postnatal depression of any severity as the outcome, two variables remained significant in the multivariable model: antenatal depression and trauma history. After controlling for trauma history, any antenatal depression was significantly associated with postnatal depression (OR 5.1; 95% CI 1.3–19.9).

## Discussion

Our results suggest that perinatal depression represents a significant disease burden among refugee and labour migrant women on the Thai-Myanmar border. Overall, 18.5% of women experienced moderate-severe depression and 39.8% experienced depression of any severity during the perinatal period. The prevalence of moderate-severe depression found in our study is in line with estimates from non-migrant women in other LMIC. For example, Woody et al. (2017) found a pooled antenatal prevalence of 19.2% and postnatal prevalence of 18.7% in low-income settings [3]. Our estimates are also in line with findings from a meta-analysis of migrant women from LMIC who resettled in HIC, which reported pooled estimates of 17% for major depressive disorder and 31% for any depressive disorder [13]. The finding that over one in three women in our setting exhibited depressive symptoms during the course of her pregnancy and post-partum period is worrying, especially in light of the serious and potentially long-lasting consequences of perinatal depression to women, their children and society at large. The relatively high incidence of depression during the course of pregnancy and the early post-partum period – 15.4% for moderate-severe depression and 28.5% for any depression – highlights the need for healthcare workers to remain vigilant for new-onset symptoms of depression during this period. The slight increase in incidence between the third trimester and the first month post-partum suggests that the time immediately preceding and following delivery may be a time at which women are particularly prone to developing the condition.

The slight decline of both moderate-severe depression and any depression prevalence from the first trimester of pregnancy through to the end of the pregnancy is consistent with findings from other studies [23]. However, this trend must be interpreted with caution given that in our study, women identified as having depression were offered treatment which may have affected prevalence estimates at subsequent assessment times. Overall, the percentage of participants who received treatment was small, at less than 10% of the total sample. But even amongst women who were not treated, the repeated SCID interviews in and of themselves may have had a therapeutic effect by providing women with an opportunity to talk about symptoms in an open manner to a healthcare professional – an opportunity which in this setting is rare [24]. Depression of any severity was more likely to persist over the course of the perinatal period than depression of moderate-severe severity. Again, these results must be interpreted cautiously given the treatment of women with depression. However, the trend suggests that there may be an aspect of milder depressive symptoms – which are included in the ‘any’ depression category but not in the moderate-severe depression category – that are less amenable to treatment. This might be explained by milder symptoms of depression being reflective of on-going difficult life circumstances – which are unchanging over time in this setting – rather than of more severe underlying psychopathology.

The decision to include the less severe DSM-IV category of Depression ‘Not Otherwise Specified’ (NOS) was based on a number of factors. First, we felt it was important to capture the full epidemiological burden of distress within this population, and including depressive symptoms across the entire spectrum of severity was deemed important to ensuring all cases were identified. Second, despite it being regarded as less clinically meaningful than Minor and Major Depressive Disorders, Depression NOS can nevertheless cause significant emotional distress and impact negatively upon individuals’ social and occupational functioning. The many women who fell into this category in our setting, along with the persistence of their symptoms over time and the impact of these symptoms on their everyday lives suggest that this category represents a clinically important state of distress which cannot be ignored. Excluding this category from data collection or data analysis risks falsely labelling these women as psychologically unaffected by their circumstances, fails to recognise the true extent of the burden of depression in this setting and risks leaving a considerable number of women less able to access support.

Risk factors differed between moderate-severe depression and any depression. For moderate-severe perinatal depression, the main risk factors identified were psychosocial, including interpersonal violence (OR 4.5), a history of trauma (OR 2.4), a self-reported history of depression (OR 2.3), perceived insufficiency of social support (OR 2.1) and labour migrant (as opposed to refugee) status (OR 2.1). Low levels of social support have consistently been associated with perinatal depression across multiple and diverse settings, and it is unsurprising that this was one of the key factors associated with depression in our population [8, 10, 14]. In this context of geo-political tension, women often live in separation from family members who have remained in home towns or villages in Myanmar or resettled abroad. The support networks normally available to these women from extended family members is therefore often lacking. Associations between interpersonal violence and perinatal depression are also well-documented: an important finding given the increased occurrence of interpersonal violence during pregnancy [25]. Similarly, experiences of trauma are common among many migrant populations, and mechanisms to sensitively assess and manage trauma histories are called for as part of a wider assessment of mental well-being [26]. The relevance of a self-reported history of depression is of particular interest. On the Thai-Myanmar border, mental disorders are rarely formally diagnosed by medical professionals. The strong association we found between women’s own subjective accounts of prior experiences of depression suggests that asking women directly about depressive histories may be useful as part of a wider programme to detect perinatal depression in this setting.

In the analysis of factors associated with *any* depressive disorder – i.e. including the milder category of Depression NOS – interpersonal violence (OR 4.4), a history of depression (OR 3.4) and experience of trauma (OR 2.2) remained important risk factors. However, telephone ownership (OR 1.6) was also significantly associated, and an additional member of the household employed (OR 0.57) was a significant protective factor. These variables were included as proxies for household income (telephone ownership) and income stability (additional household member working). Their significance in the multivariable model suggests that when milder experiences of depression are included in the outcome, socio-economic factors come into play, as these impact heavily upon the daily lives of migrant and refugee women in this setting. This adds weight to the idea suggested above that the lower-severity depressive episodes are reflective of general life situations rather than an endogenous depression. If this is indeed the case, moderate-severe perinatal depression and perinatal depression of any severity represent distinct diagnostic categories, each important in its own right. Finally, the magnitude of the association between antenatal and postnatal depression (OR 5.1) was striking and emphasises the importance of identifying and supporting pregnant women with depression as early as possible to avoid persistence of the condition postnatally.

We found that labour migrants had a higher likelihood of depression than refugees. Although both populations endure hardships, labour migrants may arguably be more vulnerable to day-to-day stressors such as securing an income and living under the constant threats conferred by their undocumented status. In a study of labour migrants in the urban centre of Mae Sot, forced overtime labour, verbal abuse and withholding of documents and salaries were common and linked to depression [12]. Refugees – though they face multiple other psychological stressors – have at least some of their basic needs such as a minimal ration of food per family and housing requirements met within the camp setting, and thus may be slightly less at risk of depression relative to their labour migrant counterparts.

### Strengths and limitations

To our knowledge, this represents the first cohort study of perinatal depression among labour migrant and refugee women in a LMIC setting. Our findings offer a direct comparison of labour migrants and refugees within the same setting. Few other studies have included different categories of migrants, and those that did have not always conducted comparative analyses [10]. Hard-to-reach migrants including those with insecure legal status and those who do not speak the host country language – such as the women included in this study – have rarely been included in research to date, and the importance of this group being represented has been emphasized [14]. The use of a diagnostic rather than screening instrument to identify women with depression constitutes a further strength, enabling greater accuracy and addressing a significant gap in the existing evidence, which relies heavily on screening tools [13]. Our study adopted a comprehensive approach to perinatal depression: outcomes encompassed the rarely studied category of Depression NOS, while inclusion of a wide range of socio-demographic, psychosocial and clinical exposures enabled the effects of multiple potential risk factors to be explored simultaneously. The longitudinal design allowed associations between antenatal and postnatal depression to be examined. Finally, given that over 90% of migrant women in this region access antenatal care, we believe that the results are representative of the local migrant population [27].

There were also a number of limitations. The SCID may have missed culture-specific manifestations such as somatic symptoms which are common in Asian cultures [4, 28]. This could have led to an under-estimation of the true prevalence of perinatal depression. However, previous work on the Thai-Myanmar border found that the SCID elicited more information than a number of screening tools [29, 30]. Working across different languages introduces a risk of misinterpretation and loss of meaning. Social desirability bias and stigma may have influenced women’s disclosure of symptoms [24]. Due to resource constraints, some interviews were conducted by trained counsellors rather than a physician. However, the ability of local counsellors to establish trust, understand, recognise and respond to participating women can be regarded as a considerable strength. Furthermore, in resource-constrained settings it is neither desirable nor sustainable to rely on specialists to identify or manage mental disorders and indeed, training and engaging local staff in this study built up experience and expertise within the community and ensured its sustainability.

The treatment of women who were diagnosed with depression introduces uncertainty into the point prevalence estimates of depression from the second trimester of pregnancy onwards, as the symptoms of those who were followed-up and treated may have resolved as a result and led to an under-estimation of natural rates. It also meant that the duration of depressive episodes could not be ascertained – a potentially significant omission when one considers, for instance, that depression which lasts for the whole pregnancy differs considerably from depression that starts at the end of pregnancy and ends at birth. However, we felt strongly that *not* treating women experiencing depression would not have been ethical practice and it was important for all those participating in the study to feel supported. The point prevalence at baseline (first trimester of pregnancy) remains unaffected, and the period prevalence also resolves this issue to a degree, as any woman who experienced depression at any timepoint (even if it subsequently resolved following treatment) is accounted for in this measure. Nevertheless, we cannot rule out the possibility that treatment of participants may have led to the under-estimation of true prevalence.

The lack of a non-migrant control group makes it difficult to establish to what extent depression was associated with migrant status rather than general socio-economic adversity. The political tensions in this region meant it was not possible to recruit non-migrant Burmese or Karen women living in comparable regions of Myanmar, Although the overall sample size was sufficient for our study aims, the smaller number of women who received treatment meant that the effectiveness of counselling and medication offered could not be reliably assessed, nor could a stratified analysis according to treatment status be conducted. Finally, the inclusion of a large number of co-variates in logistic regression may have increased the probability of type II errors. However, the fact that risk factors identified in this study are highly plausible and well-aligned with findings from other settings lends credence to the validity of the methods and final model.

The Thai-Myanmar border region is a resource-constrained area with diverse health priorities, and recommendations for practice must remain feasible and realistic within the context of this environment. Evidence from HIC suggests some benefit of primary prevention efforts such as strengthening social networks, women’s empowerment and addressing interpersonal violence [31]. The effectiveness of such efforts in LMIC needs evaluating. Secondary prevention efforts for the early detection of perinatal depression may also be indicated [28]. The perinatal period offers a valuable window of opportunity of frequent contact between women and health providers, and routine screening of all pregnant women is recommended across many HIC settings. Optimal timing of screening initiatives needs to be established, especially in light of continued onset of new cases throughout the perinatal period. Ongoing staff training and regular debriefing opportunities are essential for long-term sustainability. Finally, effective and integrated treatment and referral mechanisms are required. The evidence base around effective interventions in LMIC settings is growing. Initiatives delivered by trained non-specialists have been well-received, and embedding programmes within the community can reduce the stigma of attending [31]. Ultimately, addressing perinatal depression in this setting requires long-term, multi-sectoral collaborations at community, regional and national levels to tackle the wider socio-economic determinants.

## Conclusion

This research highlights the significant burden of perinatal depression among migrant women on the Thai-Myanmar border, with as many as one in three women experiencing the condition during pregnancy or the first month post-partum. With drivers of migration showing no signs of calming, sustainable and effective methods to identify and treat perinatal depression in migrant populations are urgently needed. Improving the recognition and management of perinatal depression requires long-term collaborative and cross-sectoral working – a hard task in low-income, migrant situations where political tensions and instability are the norm. Efforts to better manage perinatal depression must be accompanied by preventative measures to promote mental health and address the wider, social determinants of health. It is only by tackling the complex array of factors impacting upon women’s mental health – from experiences of trauma through generalised poverty and unemployment to abusive relationships – that the plight of these communities might be improved.

## Data Availability

The dataset generated for this study is not publicly available due to the sensitive nature of the information and the vulnerable legal status of the participants. Data can therefore only be accessed by submission of a request to the Data Access Committee at Mahidol Oxford Tropical Medicine Research Unit (MORU). The data sharing policy is available at http://www.tropmedres.ac/data-sharing. Please send requests to Dr. Phaik Yeong Cheah (phaikyeong@tropmedres.ac).

## Abbreviations

ANC: Antenatal clinic
CI: Confidence interval
DSM-IV: Diagnostic and Statistical Manual of Mental Disorders, 4th edition
EGA: Estimated gestational age
HIC: High income countries
LMIC: Low- and middle-income countries
NOS: Not otherwise specified
OR: Odds ratio
SCID: Structured Clinical Interview for the Diagnosis of DSM-IV Disorders
SMRU: Shoklo Malaria Research Unit
